# Early-Life Factors and Risk of Parkinson’s Disease: A Register-Based Cohort Study

**DOI:** 10.1371/journal.pone.0152841

**Published:** 2016-04-15

**Authors:** Bojing Liu, Honglei Chen, Fang Fang, Annika Tillander, Karin Wirdefeldt

**Affiliations:** 1 Department of Medical Epidemiology and Biostatistics, Karolinska Institutet, Stockholm, Sweden; 2 Epidemiology Branch, National Institute of Environmental Health Sciences, Research Triangle Park, North Carolina, United States of America; 3 Department of Clinical Neuroscience, Karolinska Institutet, Stockholm, Sweden; New York City Department of Health and Mental Hygiene, UNITED STATES

## Abstract

Parkinson’s disease (PD) may take decades to develop and early life exposures such as infection may be important. However, few epidemiological studies have evaluated early life risk factors in relation to PD risk. We therefore examined such associations in a prospective analysis of 3 545 612 individuals born in Sweden between 1932 and 1970 without PD on January 1, 2002. Incident PD cases were identified using the Swedish Patient Register during 2002–2010. Information on sibship size, number of older and younger siblings, multiple births, parental age, birth month and season was obtained from the Swedish Multi-Generation Register. Monthly data on national burden of influenza-like illness during 1932–1970 were obtained from the Swedish Public Health Agency. Hazard ratios with 95% confidence intervals (CI) were estimated using Cox proportional hazards regression. During the follow-up, 8779 incident PD cases were identified. As expected, older age, male sex, parental occupation as farmers, and family history of PD were associated with higher PD risk. Overall, early life factors, including flu burden in the year of birth, were not associated with PD risk, although we did find a lower PD risk among participants with older siblings than those without (HR = 0.93, 95%CI: 0.89, 0.98). Our study therefore provided little support for important etiological contributions of early life factors to the PD risk late in life. The finding of a lower PD risk among individuals with older siblings will need confirmation and further investigation.

## Introduction

Parkinson’s disease (PD) is an age-related neurodegenerative disorder affecting 1% of the population over 60 years and up to 4% of those older than 80 years [1]. The etiology of PD remains largely unknown. Recent genome-wide association studies have identified over two dozen genomic susceptibility loci for late-onset sporadic PD, but these may collectively account for only a small proportion of all cases [2].

PD may take decades to develop and a wide range of non-genetic factors may come into play in at various stages of disease development [3]. Numerous epidemiological studies have reported associations of PD with mid or late-life factors such as smoking, coffee drinking, and exposures to pesticides[3]. However, few studies have evaluated potential roles of early life factors in PD development primarily due to the difficulty in exposure assessment. [4]. On the other hand, the hypothesis that early life factors may contribute to PD late in life is appealing. The pre- and postnatal periods are vital time spans for brain development, during which the generation, migration and proliferation of neurons is completed, and the fundamental structure of the brain is established [5]. In support of this hypothesis, endotoxin injection into gravid rats induced dopamine neuron loss among new births, indicating that prenatal infection might contribute to PD development [6].

We therefore took advantage of several nationwide registers and historical data in Sweden and investigated three developmental aspects of PD etiology: 1) early life infection related factors including sibling structure, birth month and season.2) parental age at birth, and 3) multiple births. In addition, we performed an ecological analysis evaluating the impact of flu burden in the year of birth on PD risk.

## Materials and Methods

### Swedish population and health registers

The present study was conducted using multiple nationwide Swedish registers cross-linked through the unique national personal identification numbers assigned to all Swedish residents. The *Swedish Multi-Generation Register* (MGR) contains information about biological and adoptive parents for persons who were born in 1932 or later, and were alive and lived in Sweden in 1961. The MGR covers more than 95% of Swedish born residents and more than 22% of foreign-born residents in Sweden [7]. The Swedish *Patient Register* was first established in 1964/1965 to collect inpatient discharge records and became nationwide in 1987 [8]. From 2001, the Patient Register was expanded to collect >80% hospital records of outpatient visits; data from private caregivers were not included, while the coverage of outpatient data from public caregivers is almost 100% [9]. Thus, since 2001, the Patient Register has recorded information on each inpatient visit and vast majority of the outpatient hospital visits of all Swedish residents, including dates of each visit, primary diagnosis, and up to 21 secondary diagnoses that are coded according to the Swedish revisions of the International Classification of Diseases (ICDs). In addition to the MGR and Patient Register, we also linked our register data to the Migration Register and Swedish Population and Housing Censuses in 1960, 1970, 1980, and 1990 that contain information on socio-economic status [10].

### Identification of PD patients

We defined an individual as a PD case if there was a hospital inpatient or outpatient record with PD either as the primary or secondary diagnosis from 1964 onward. We used the ICD codes 350, 342, 332.0 and G20 from ICD7, ICD8, ICD9 and ICD10, respectively to identify PD. The date of first hospital discharge or outpatient contact was used as the date of diagnosis. The validity of PD diagnoses from the inpatient register has been validated against detailed clinical evaluation and showed a positive predictive value (PPV) for any PD diagnosis (primary or secondary) of 70.8%, which increased to 83.0% when only primary diagnosis was considered. [11] The validity of PD diagnoses from the outpatient register has yet to be validated.

### Study design

**Fig 1:**
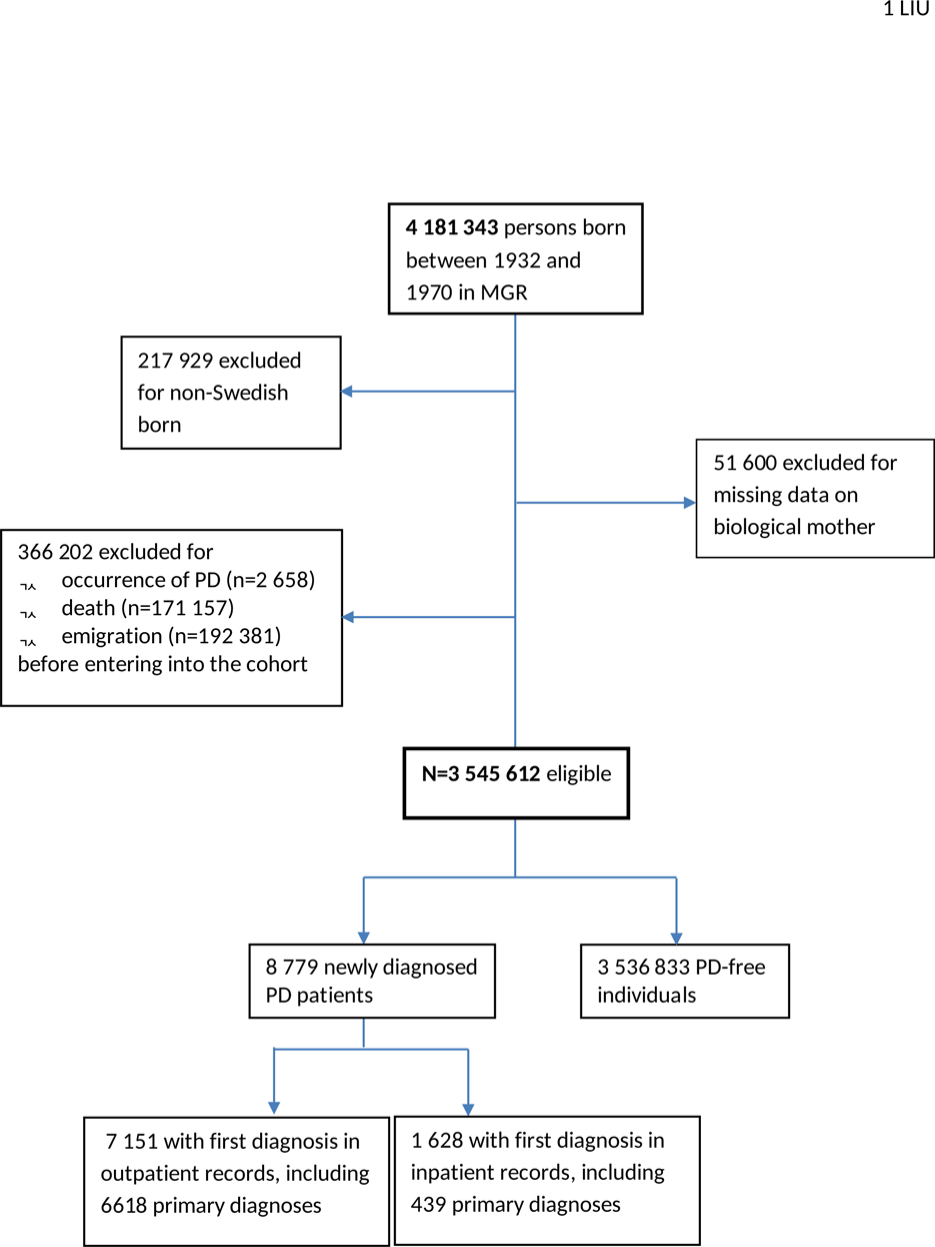
Study population within the Swedish Multi-generation Register (MGR). The study population included individuals who were born in Sweden during 1932–1970, with available information on maternal links in the MGR, alive and free of PD, lived in Sweden, and were 40 years or older on January 1, 2002, or turned 40 years during the study period.

The present study included 3 545 612 unique persons who satisfied the following inclusion criteria: born in Sweden between 1932 and 1970, available information on maternal links in the MGR, alive and free of PD on January 1, 2002, and 40 years or older on January 1, 2002 or turned 40 years during the study period (Fig 1). As the outpatient records were not available until 2001, we suspected that cases from the first year of outpatient data were more likely to be prevalent cases than those identified in later years. We therefore started follow-up on January 1^st^ 2002, one year after the initiation of Outpatient Register. Incident PD cases after January 1, 2002 were identified using the code G20 from ICD10. We defined end of follow-up as the date of PD diagnosis, death, emigration out of Sweden, or December 31, 2010, whichever occurred first.

#### Assessment of birth characteristics

Siblings of an index person were defined as either a full sibling or maternal half sibling. We did not consider paternal half siblings, assuming that maternal siblings are more likely to live in the same household and therefore share more early life environmental exposures compared to paternal half siblings. Based on all siblings identified, we derived the following variables: sibship size (1, 2, 3, or ≥4); number of older siblings (0, 1, 2, or ≥3); number of younger siblings (0, 1, 2, or ≥3); birth interval between an index person and their nearest older and younger siblings (<2, 2–6, or >6 years); maternal and paternal age at birth (≤20, 21–25, 26–30, 31–35, 36–40, or >40 years); multiple births (yes or no); birth month; and birth season (spring: March-May, summer: June-August, autumn: September-November, winter: December-February).

#### Family history of PD and parental socio-economic status

Family history of PD was defined as having at least one first-degree relative (full sibling, parent or offspring) diagnosed with PD. For all cohort members, we first identified first-degree relatives from the MGR; the relatives were then linked to the Patient Register to identify a diagnosis of PD.

Parental socio-economic status was defined based on the Swedish socio-economic index (SEI) that distinguishes employers from employees, and further classifies employees into various social classes [12]. Information on SEI was obtained from the Swedish Population and Housing Censuses that were conducted every five years from 1960 to 1990 by Statistics Sweden [10], and were mandatory for Swedish residents 15 years or older. Of the 1960 and 1970 censuses, we considered the one that was closest to the birth year of the index person. Missing information in any of the 1960 or1970 censuses was replaced by information from the other census whenever possible. Parental socio-economic status was defined as the highest socioeconomic status of the parents’, and categorized as farmers, blue-collar employees, white-collar employees, self-employed, and unclassifiable.

#### Assessment of Influenza-like illness activities in the year of birth

Starting in 1911, general practitioners in most parts of Sweden were required to report influenza-like illness (ILI) occurrences on a monthly or weekly basis to the Swedish Public Health Agency. The adherence of the report was generally high [13], We obtained yearly national data on ILI from 1932 to 1970s. During this period, we calculated the annual burden of ILI by dividing the number of ILI cases by the corresponding mid-year population size in each year [14], ILI burden was first used as continuous variable and subsequently as categorical variable (low, intermediate, and high burden using the cut-offs of 500/100 000 and 1500/100 000 person-years).

### Statistical analysis

We used Cox proportional hazard regressions to estimate hazard ratios (HRs) and 95% confidence intervals (CIs), with attained age as the underlying time scale and robust standard error accounting for the relatedness in our study population. We conducted both univariable and multi-variable analyses adjusting for sex, birth year categories (1932–1941, 1942–1951, 1952–1961, and 1962–1970), family history of PD, and parental SEI. Only variables statistically significantly associated with PD risk in the univariable models were adjusted for in the multivariable analyses. Wald test was used to calculate two-sided p-values of the overall association between each categorical variable and PD outcome. The proportional hazard assumption was evaluated for each independent variable in the model. As the assumption was violated for PD family history, we derived estimates for all exposure variables stratified by the family history of PD. The overall goodness-of-fit of the Cox models was tested using the method suggested by Gronnesby and Borgan [15]. In addition to the primary analyses, we also conducted sensitivity analyses including cases for which PD was noted as the primary diagnosis. We used Stata 13 for statistical analyses with 2-sided alpha of 0.05. The study was approved by the Regional Ethics Committee in Stockholm. Since this was a register-based study, it was not possible to obtain written informed the consent by all the participants. Individual information was anonymized and de-identified prior to analysis.

## Results

A total of 8 779 PD cases were identified during the follow-up (27.1 million person-years), mostly (n = 7 057) with PD as the primary diagnosis. The incidence rate (per 100 000 years) was 32.4 overall, 39.5 for men and 25.2 for women. Further, the increase increases with age: 96.5 for those born in 1932–1941, 31.1 for 1942–1951, 8.1 for 1952–1961, and 4.1 for 1962–1970. The average age at PD diagnosis (±SD) was 65.1 (±7.6) years. As expected, being a male, born to a farming family, and having a family history of PD were all associated with a higher risk for PD (Table 1).

**Table 1:** Characteristics of the Swedish Population Cohort Born in 1932–1970.

	Study population No. (%)	PD cases No. (%)^1^	Adjusted for attainted age		Adjusted for multi-variables^2^	
			HR	95% CI	HR	95% CI
**Total**	3 545 612 (100.0)	8 779 (0.25)				
**Sex**						
Women	1 746 866 (49.27)	3 391 (0.19)	ref		ref	
Men	1 798 746 (50.73)	5 388 (0.30)	1.64	1.57, 1.71	1.66	1.58, 1.73
**Birth year category**						
1932–1941	613 998 (17.32)	5 009 (0.82)	ref		ref	
1942–1951	1 049 644 (29.60)	2 860 (0.27)	0.89	0.82, 0.96	0.92	0.84, 1.00
1952–1961	952 357 (26.86)	735 (0.08)	0.81	0.69, 0.95	0.82	0.70, 0.97
1962–1970	929 613 (26.22)	175 (0.02)	0.95	0.70, 1.30	0.98	0.72, 1.34
Wald test *P* value				**<0.01**		**0.04**
**Parental socio-economic status**						
Farmers	448 020 (12.64)	1 618 (0.36)	1.11	1.04, 1.18	1.10	1.03, 1.17
Blue-collar employees	1 248 382 (35.21)	2 700 (0.22)	1.02	0.92, 1.13	1.03	0.98, 1.09
White collar employees	1 543 915 (43.54)	3 165 (0.20)	ref		ref	
Self-employed	67 707 (1.91)	167 (0.25)	1.00	0.85, 1.17	0.98	0.84, 1.15
Unclassifiable	102 309 (2.89)	451 (0.44)	1.02	0.92, 1.13	1.03	0.93, 1.14
Missing	135 279 (3.82)	678 (0.50)				
Wald test *P* value				**0.02**		**0.05**
**Family history of PD**						
No	3 278 886 (92.48)	7 450 (0.23)	ref		ref	
Yes	135 046 (3.81)	754 (0.56)	1.86	1.72, 2.02	1.88	1.74, 2.04
Missing	131 680 (3.71)	575 (0.44)				

1. Percentages were calculated by dividing the number of study population in each category by the corresponding number of PD cases.

2. Model included sex, birth year category, parental SES status, and family history of PD.

Most early life factors studied were not associated with PD risk (Table 2). The only exception was whether or not the participants had older siblings. Compared to those without, PD risk was 7% lower among participants with older siblings (HR = 0.93, 95%CI: 0.89, 0.98). There was however no further associations with the number of older siblings or the interval length between the index persons and their older siblings. The risk estimates were essentially the same in the sensitivity analysis where only cases with a primary diagnosis of PD were included (data not shown).

**Table 2:** Birth Characteristics, Parental Age, and PD Risk.

	Study population	PD cases	Adjusted for attainted age		Adjusted for multi-variables^1^	
	No. (%)	No. (%)	HR	95% CI	HR	95% CI
**Total**	3 545 612 (100.0)	8 779 (0.25)				
**Sibship size**						
1	469 008 (13.23)	1 874 (0.40)	ref		ref	
>1	3 076 604 (86.77)	6 905 (0.22)	0.99	0.94, 1.04	0.96	0.91, 1.02
# of sibship size						
2	1 227 553 (34.62)	2 717 (0.22)	0.99	0.93, 1.05	0.97	0.91, 1.04
3	940 925 (26.54)	1 929 (0.21)	0.98	0.92, 1.05	0.96	0.90, 1.03
> = 4	908 126 (25.61)	2 259 (0.25)	0.99	0.93, 1.06	0.95	0.89, 1.02
Wald test *p*-value				0.95		0.57
**Older siblings**						
0	1 688 407 (47.62)	5 384 (0.32)	ref		ref	
≥1	1 857 205 (52.38)	3 395 (0.18)	0.97	0.93, 1.01	0.93	0.89, 0.98
# of older siblings						
1	1 094 487 (30.87)	2 144 (0.20)	0.95	0.90, 1.00	0.92	0.87, 0.97
2	463 344 (13.07)	813 (0.18)	1.03	0.95, 1.11	0.98	0.90, 1.06
> = 3	299 374 (8.44)	438 (0.15)	0.97	0.88, 1.07	0.91	0.82, 1.01
Wald test *P* value				0.14		**0.01**
**Birth interval from the nearest elder sibling (years)**						
No elder siblings	1 688 407 (47.62)	5 384 (0.32)	ref		ref	
<2	404 456 (11.41)	931 (0.23)	0.94	0.88, 1.01	0.91	0.84, 0.98
2–6	1 133 417 (31.97)	2 047 (0.18)	0.98	0.93, 1.04	0.94	0.89, 1.00
>6	319 332 (9.01)	417(0.13)	0.95	0.86, 1.05	0.93	0.84, 1.04
Wald test *P* value				0.37		**0.02**
**Younger siblings**						
0	1 512 610 (42.66)	3 471 (0.23)	ref		ref	
≥1	2 033 002 (57.34)	5 308 (0.26)	1.01	0.96, 1.05	1.00	0.96, 1.05
# of younger sibling						
1	1 151 339 (32.47)	2 685 (0.23)	1.02	0.97, 1.07	1.01	0.96, 1.07
2	541 666 (15.28)	1 426 (0.26)	1.01	0.95, 1.08	1.02	0.95, 1.09
> = 3	339 997 (9.59)	1 198 (0.35)	0.98	0.92, 1.05	0.96	0.90, 1.03
Wald test *P* value				0.82		0.52
**Birth interval from the nearest younger sibling (years)**						
No younger siblings	1 512 610 (42.66)	3 471 (0.23)	ref		ref	
<2	407 997 (11.51)	1 129 (0.28)	1.00	0.93, 1.07	0.99	0.92, 1.06
2–6	1 234 920 (34.83)	3 035 (0.25)	1.01	0.96, 1.06	1.00	0.95, 1.06
>6	390 085 (11.00)	1 144 (0.29)	1.01	0.94, 1.08	1.02	0.95, 1.09
Wald test *P* value				0.97		0.94
**Maternal age category (years)**						
= <20	417 882 (11.79)	749 (0.18)	1.00	0.92–1.09	1.01	0.93, 1.11
21–25	1 036 476 (29.23)	2 232 (0.22)	ref		ref	
26–30	998 216 (28.15)	2 583 (0.26)	1.06	1.00, 1.12	1.05	0.99, 1.11
31–35	658 844 (18.58)	1 882 (0.29)	1.06	1.00, 1.13	1.04	0.97, 1.11
36–40	340 067 (9.59)	1 042 (0.31)	1.08	1.00, 1.16	1.05	0.97, 1.14
>40	94 127 (2.65)	291 (0.31)	0.99	0.88, 1.12	0.98	0.85–1.13
Wald test *P* value				0.17		0.55
**Paternal age category (years)**						
= <20	109 291 (3.08)	131 (0.12)	0.89	0.75, 1.07	0.91	0.76, 1.09
21–25	657 842 (18.55)	1 206 (0.18)	1.01	0.94, 1.08	1.02	0.95, 1.09
26–30	969 389 (27.34)	2 193 (0.23)	ref		ref	
31–35	803 072 (22.65)	2 144 (0.27)	1.04	0.98, 1.11	1.03	0.97, 1.09
36–40	502 775 (14.18)	1 404 (0.28)	1.03	0.96, 1.10	1.01	0.95, 1.09
>40	369 333 (10.42)	1 111 (0.30)	1.04	0.97, 1.12	1.03	0.95, 1.11
missing	133 910 (3.78)	590 (0.44)				
Wald test *P* value				0.41		0.82
**Multiple birth**						
No	3 480 911 (98.18)	8 627 (0.25)	ref		ref	
Yes	64 701 (1.82)	152 (0.23)	0.95	0.81, 1.11	0.98	0.83, 1.16

1 Multi-variable adjusted models included sex, birth year category, parental SES status, and stratified by family PD history.

**Fig 2:**
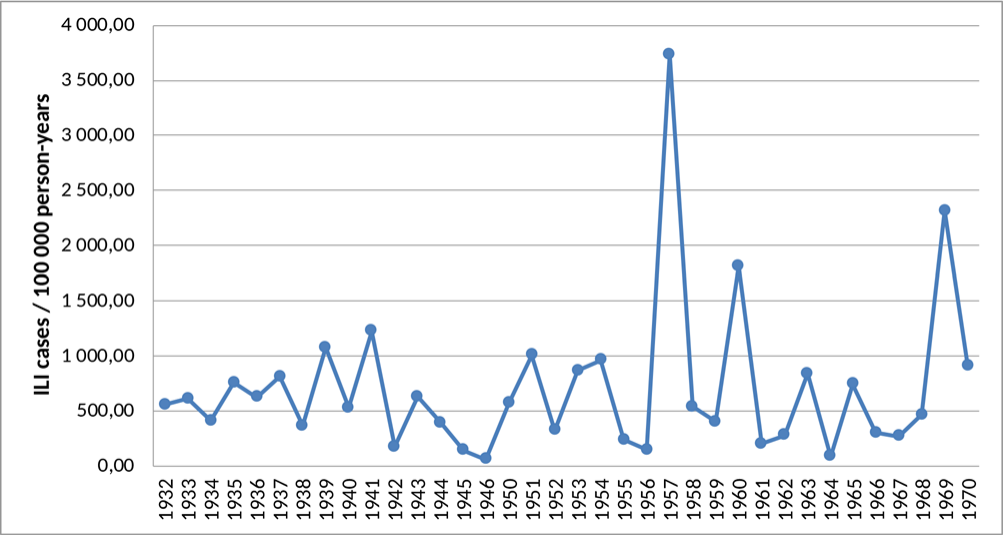
Incidence of influenza-like illness (ILI) by year of birth. National burden of ILI in Sweden from 1932 to 1970, data in 1947–1949 were excluded due to low report rates.

In general, we did not observe any specific patterns for either month or season of birth in relation to the risk for PD (Table 3). There were three peaks of flu-like illness in Sweden between 1932 and 1970, and the year 1957 had the highest (Fig 2). In our analysis, born in these three years was not associated with a higher risk of PD later in life compared to the reference group.

**Table 3:** Birth Month, Season and Influenza-like Illness (ILI) Incidence in the Year of Birth in Relation to PD Risk.

	Study population	PD cases	Adjusted for attainted age		Adjusted for multi-variables^1^	
	No. (%)	No. (%)	HR	95% CI	HR	95% CI
**Birth month**^1^						
JAN	283 324 (7.99)	696 (0.25)	1.05	0.94, 1.17	1.05	0.94–1.18
FEB	276 384 (7.80)	706 (0.26)	1.11	0.99, 1.23	1.10	0.99–1.23
MAR	333 430 (9.40)	868 (0.26)	1.14	1.03, 1.26	1.16	1.04–1.29
APR	331 935 (9.36)	830 (0.25)	1.12	1.01–1.24	1.10	0.99–1.22
MAY	328 220 (9.26)	790 (0.24)	1.06	0.95–1.17	1.02	0.92–1.14
JUN	300 593 (8.48)	722 (0.24)	1.04	0.93–1.15	1.03	0.92–1.15
JUL	297 090 (8.38)	781 (0.26)	1.14	1.03–1.27	1.13	1.01–1.26
AUG	285 169 (8.04)	734 (0.26)	1.12	1.01–1.24	1.14	1.02–1.27
SEP	292 421 (8.25)	665 (0.23)	ref		ref	
OCT	280 629 (7.91)	700 (0.25)	1.13	1.01–1.25	1.09	0.97–1.22
NOV	262 106 (7.39)	616 (0.24)	1.06	0.95–1.18	1.05	0.93–1.18
DEC	274 311 (7.74)	671 (0.24)	1.10	0.98–1.22	1.07	0.96–1.20
Wald test *P* value				0.23		0.17
**Season**						
Spring (MAR-MAY)	993 585 (28.02)	2 488 (0.25)	1.04	0.98–1.10	1.05	0.98–1.12
Summer (JUN-AUG)	882 852 (24.90)	2 237 (0.25)	1.04	0.98–1.10	1.05	0.98–1.12
Autumn (SEP-NOV)	835 156 (23.55)	1 981 (0.24)	ref		ref	
Winter (DEC-FEB)	834 019 (23.52)	2 073 (0.25)	1.02	0.96–1.09	1.03	0.97–1.10
Wald test *P* value				0.56		0.44
**ILI category (cases/100 000 person years)**						
Low (< = 500)	1 555 345 (43.87)	3 046 (0.20)	ref		ref	
Intermediate (500–1500)	1 382 236 (38.98)	4 901 (0.35)	1.01	0.96–1.06	0.98	0.93–1.03
High (>1500)	283 580 (8.00)	117 (0.04)	1.04	0.85–1.27	1.07	0.87–1.32
missing	324 442 (9.15)	715 (0.22)	-		-	
Wald test *P* value				0.92		0.56
**Continuous ILI incidence**			1.00	1.00–1.00	1.00	1.00–1.00

1 Multi-variable adjusted models included sex, birth year category, parental SES status, and stratified by family PD history.

## Discussion

To the best of our knowledge, this is the largest and one of the first epidemiological studies to investigate early life risk factors in relation to PD risk late in life. The study took advantage of nationwide historical exposure records that were free of recall bias. We found no association of either early life family characteristics or influenza burden in the year of birth with the risk for PD late in life. The only exception was that having older siblings appeared to be associated with a lower risk for PD.

The *developmental origin of disease* hypothesis is an intriguing concept for the etiology of complex chronic diseases. According to this hypothesis, the susceptibility to adult-onset diseases may be influenced by *in utero* and neonatal environmental exposures. Evidence supporting this hypothesis includes the association between *in utero* nutrition states, coronary heart diseases, hypertension, and diabetes [16]. Recent epidemiological studies also reported that birth order and sibship size, most likely as proxies of common infections within the household, were associated with the risk of Alzheimer’s disease [17], amyotrophic lateral sclerosis [18] and schizophrenia [19].In contrast, few epidemiological studies have focused on early life environment and PD.

We were interested in the hypothesis that early life infection might be related to a higher risk for PD. In support of this, animal experimental studies showed that prenatal exposure to the endotoxin of lipopolysaccharide induce dopaminergic neuron loss in adult mice [6]. Further, an earlier report showed that individuals born in the years of influenza pandemic in the 20^th^ century had a higher risk of PD [20], although the results are not entirely consistent [21–23]. In the current study, we explored early life infection related exposures including sibship size, season and month of birth in relation to PD risk. These variables have been used in previous studies as exposures to investigate early life infections in relation to adult diseases, including Alzheimer’s disease and amyotrophic lateral sclerosis [17, 18]. In addition, we re-evaluated the proposed association between societal influenza burden in the year of birth and PD risk, using a wider spectrum of ILI data ranging from 1932 to 1970. The results of our study however did not support associations between early life infection and PD risk. The absence of association between flu burden in the year of birth and PD risk is in line with the majority of previous studies [21–23]. Our results of no association between birth month and season, sibship size and PD risk are equally consistent with previous findings [24, 25].

We did find a lower PD incidence among participants who had one or more older siblings although the interpretation of this finding is not straight forward. Previous studies have reported that history of childhood red measles and chickenpox infections were inversely related to PD risk [26]. One may speculate that this is another example of the hygiene hypothesis that early life immune challenges help develop a strong and balanced immune system later in life as exemplified allergic or autoimmune conditions such as asthma [27] and type I diabetes [28]. On the other hand, we could not exclude chance as a possible explanation as we did not find any dose-response relationship for number of older siblings or age interval between study participant and nearest older sibling. Furthermore, PD risk was not altered among those with younger siblings.

We also tested other developmental origins including parental age at birth and multiple births. The suggested mechanism underlying the paternal effect is the higher *de novo* mutation rate with increasing paternal age, which is mainly attributed to a great number of germ cell divisions [29]. The potential maternal effect, on the other hand, may be attributed to the accumulation of oxidative stress and resulting mitochondrial DNA mutations being transmitted from mothers to offspring [30]. Multiple births have been linked to preterm deliveries and low birth weight. However, results from the previous and present studies do not support the above hypotheses [24].

The current study has several strengths, including the prospective design and the exceedingly large sample size, and use of nationwide historical data. The exposure information on sibling structure, parental age, month and season of birth was retrieved from registers making the study insusceptible to recall bias. Inclusion of both prevalent and incident PD cases will not introduce reverse causality as the birth exposures are independent of PD diagnosis. However, our study also has some limitations. First, we identified potential PD patients from nationwide registers and outcome misclassification was inevitable, although previous studies validated the accuracy of PD diagnosis of inpatient visits, with a positive predictive value (PPV) of 70% among all cases and an additionally increased PPV if PD was the primary diagnosis of hospitalization (83%) [11]. Although we are yet to validate PD diagnosis in the outpatient register, it might be reasonable to assume that its accuracy is comparable to or better than that of the inpatient visits, since 92.5% of PD from outpatient visits had PD as their primary diagnosis compared to 27% from inpatient records. As a complementary analysis, we linked the patient register to the Swedish drug prescription register that records drug utilization and expenditures for prescribed drugs for the entire Swedish population since July 2005. We observed that 91.1% of the PD patients with first diagnosis in the outpatient register during 2005–2010 had been subscribed and took antiparkinsonian drugs. However, the misclassification of PD outcome is likely non-differential with regard to exposure variables. Furthermore, due to the nature of our study, sibling structure, birth season and month may be viewed as indicators of overall childhood infection levels, which might have undermined our ability to identify the association of specific early infection in relation to PD. Furthermore, although we gathered ILI data over thirty years, the intrinsic feature of ecological design of using data at integrated level limited ability to assess prenatal influenza infection to PD risk relationship. Finally, our analyses were limited to information that was recorded in the MGR and we were unable to investigate other exposures of interest; for example, birth weight or parental smoking behavior, in relation to PD risk later in life.

## Conclusions

In summary, our study provided little support for important etiological contributions of early life factors to the PD risk late in life. The finding of a lower PD risk among individuals with older siblings will need confirmation and further investigation.
